# TMD in Females with Menstrual Disorders

**DOI:** 10.3390/ijerph18147263

**Published:** 2021-07-07

**Authors:** Bożena Jedynak, Marta Jaworska-Zaremba, Barbara Grzechocińska, Magdalena Chmurska, Justyna Janicka, Jolanta Kostrzewa-Janicka

**Affiliations:** 1 Department of Prosthodontics, Medical University of Warsaw, Binieckiego str 6, 02-097 Warsaw, Poland; bozena.jedynak@wum.edu.pl (B.J.); marta.jaworska-zaremba@wum.edu.pl (M.J.-Z.); 2 University Center for Maternal and Newborn’s Health, Medical University of Warsaw, Starynkiewicza Square 1/3, 02-015 Warsaw, Poland; barbara.grzechocinska@wum.edu.pl; 3 Military Institute of Medicine, Szaserow str 128, 04-349 Warsaw, Poland; m.jedynak19@wp.pl; 4 Faculty of Medicine, Medical University of Warsaw, Żwirki i Wigury 61, 02-091 Warsaw, Poland; j.m.janicka@gmail.com

**Keywords:** temporomandibular disorder, estrogen, menstrual disorder

## Abstract

Background: Temporomandibular disorders (TMD) are a common reason for patients to present at dental offices. The majority of people with TMD are women between the age of 20 and 40 years. The purpose of this study was to assess the types and prevalence of temporomandibular disorders in female patients of reproductive age with menstrual disorders. Materials and methods: The study involved 65 females of reproductive age (18–40 years, an average of 28.00 ± 6.27 years). The women who qualified for the study were patients of the University Center for Maternal and Newborn’s Health hospitalized because of infertility or menstrual cycle disorders. Women with confirmed estrogen metabolism disorders participated in a clinical study with the use of Diagnostic Criteria for Temporomandibular Disorders (DC/TMD). Results: In the studied female patients with menstrual disorders, temporomandibular disorders (92.3%) were frequent occurrences. The most common type was intra-articular joint disorders (68%). Other reported complaints included masticatory muscle pain (44.62%), and degenerative joint diseases (12.3%). Conclusions: 1. In women with menstrual disorders, TMD may exist. 2. In women with TMD symptoms, their medical history should be extended to include the diagnosis of female hormone disorders.

## 1. Introduction

Temporomandibular disorders (TMD) are prevalent in the adult population. In recent decades, based on clinical observation, there has been an increase in the number of cases of TMD [1]. The literature data presents various epidemiological values concerning TMD prevalence [1,2,3,4]. Studies by List et al. demonstrated that 15% of adults and 7% of adolescents were affected by the condition [5], contrary to Valesan et al. who reported TMD prevalence at 31% in the population of adults/the elderly and 11% in children/teenagers [6]. These disorders affect the whole adult population but the highest number of diagnosed cases is reported in the 20–40 years age range [1,5,7,8,9,10]. At the same time, women are reported to be more often affected by masticatory dysfunctions than men. Women are two-to-four times more likely to experience pain due to masticatory activities than men [1,2,4,7,8,9,10,11,12,13,14,15,16,17,18,19]. Besides a high incidence of TMJ and/or muscle dysfunctions in women, they also suffer from a wider range of symptoms and their increased intensity [14,20]. These disorders may affect temporomandibular joint (TMJ) structures (intra-articular disorders and joint degenerative disease), masticatory muscles, and both masticatory muscles and the TMJ [21,22,23]. The clinical symptoms of TMD are manifested by disturbed movement of the mandible (improper range and pattern), the presence of acoustic manifestations (clicks, crepitations) in the TMJ during mandibular movement, pain in the TMJ, either spontaneous or provoked by consumption of food, and lastly pain in the masticatory muscles, the head, and the neck. All of the above-mentioned manifestations may compromise the performance of basic daily activities and lower the patient’s quality of life [5,7,8,11,19,24]. The most frequent TMD complaints from patients presenting at dental offices are painful sensations and the presence of acoustic manifestations. Due to a wide range of symptoms of these disorders and their location, patients with pain seek the assistance of non-dental specialists, including ENT doctors, neurologists and even psychiatrists. On the other hand, a meticulously conducted clinical dental examination may facilitate the diagnosis of systemic diseases [25]. Multidisciplinary treatment of temporomandibular diseases magnifies the bone base and increases its quality, which improves the conditions for the use of dental implants [26,27]. The aetiology of TMD is multifactorial, complex, and difficult to diagnose, with an insufficiently studied pathomechanism. A number of biological and psychosocial risk factors for TMD development have been identified. These include: occlusal dysfunctions, chronic stress, parafunctions, individual tendency to excessive articular flaccidity, trauma, age, gender, and genetic or anatomic determination [5,6,7,8,10,15,18,19,20,28]. Occlusal abnormalities are listed first among the local factors predisposing to the development of TMD, although their role in the pathomechanism of TMD is ambiguous and sometimes controversial. Cancaglini et al. assessed the distribution of occlusal contacts in patients with TMD in comparison with the control group. They observed a greater number of occlusal contacts in people with unilateral TMD on the side of the diseased TMJ joint compared to healthy patients [29]. Clinical observations show that the implementation of orthodontic treatment helps to relieve TMD pain by restoring the harmony of the occlusion between the masticatory muscles and TMJ activity [30]. Retention devices used after orthodontic treatment, whose task is to maintain the corrected occlusion, play an invaluable role [31].

Even though the influence of genetic basis on TMD etiopathogenesis is poorly known, there are two polymorphic genes: 5-HTT (serotonine transporter-“happiness hormone”) and COMT (catechol-O-methyltransferase), which are linked to TMD prevalence. Their polymorphism predisposes to TMD, increases the susceptibility to the severity of the perceived pain and the transition to chronic pain phase as well as increases the tendency to depression and/or anxiety [32]. An adequate lifestyle, emotions, and other environmental (epigenetic) factors may trigger expression of these genes. The latest studies indicate that there are no specific genes that can directly influence the onset or duration of pain in TMD patients [33]. Today, the best-documented factors having a destructive effect on the masticatory organ related to civilization stress. Its presence leads to increased tension and activity of masticatory muscles [20,34,35]. It has to be remembered that TMD rarely constitute an isolated, single disease entity. Systemic diseases may modulate the course of TMD (onset, severity, duration, and response to therapy).

A hybrid concept of the aetiology of these disorders indicates parallel effects of many factors, both local and systemic, which contribute to the development of dysfunction symptoms within the masticatory organ. Therefore, it is recommended to use noninvasive and reversible methods in the first phase of the treatment of symptoms of the masticatory organ dysfunction. This is of utmost importance, since there are cases of spontaneous remission of symptoms and also of the effects of patients’ stressful and psycho-emotional condition on the disease picture. Bearing this in mind, it is recommended that the treatment should focus on muscle relaxation and unloading of joint structures with parallel action of pain relievers and anti-inflammatory drugs. Nowadays, physiotherapy plays an important role, as do dry needle techniques [36,37]. The currently accepted method of TMJ pain treatment in cases of internal dysfunctions is intraarticular injection of hyaluronic acid and platelet-rich plasma (PRP) for regeneration and revascularization [38,39]. The occurrence of TMD in postpubertal women and in women of reproductive age, and their decrease following menopause, may indicate the involvement of female sex hormones in the aetiology of these disorders [34]. Since TMD predominate in women, the role of female sex hormones has become the subject of many studies [13,20,32,34,35,40,41,42,43]. Estrogens and progesterone in blood are bound with plasma proteins and impact the metabolism of other tissues and organs. Estrogens may exert their influence on cells by means of several mechanisms, such as a direct or indirect effect on gene expression via nuclear estrogen receptors as well as receptors present in cell membranes. Peripheral action consists in, among others, modulating the immunological response via receptors in the thymus, the marrow or the spleen. Estrogens may also affect monocytes and macrophages regulating the production of proinflammatory cytokines (interleukin-1, interleukin-2, TNF-α). Genetic variants of the receptors’ structures may result in a variety of clinical outcomes including joint inflammations, bone lesions and pain. The proven anti-inflammatory and analgesic effect of estrogen is related to its antinociceptive activity dependent on the central secretion of opiates [44,45]. On the other hand, the elevated estrogen levels may provoke stronger stimulation of nociceptive endings of the trigeminal nerve, increased masticatory muscle tension and central sensitization. This is manifested with increased response to traumatic stimulus.

### Aim of the Study

The aim of the study was to determine the type and frequency of TMD in female patients with menstrual disorders aged 18–40 years.

## 2. Material and Methods

### 2.1. Study Group

In this study, 65 females with menstrual disorders and 61 age-gender—matched healthy controls participated. The total of 65 females aged 18–40 years (mean 28.00 ± 6.27 years) were patients of gynecological wards who were hospitalized in 2019 at the University Centre for Maternal and Newborn’s Health in Warsaw for the purpose of treatment of menstrual disorders and/or infertility. The inclusion criterion was female reproductive hormone disorders (estrogen and/or progesterone), implicated in menstrual disorders according to groups (subgroups) categorized by WHO. The patients expressed their informed consent for the participation in the study and fully co-operated.

Group I comprised women with hypogonadotropic hypogonadism. Concentrations of FSH, LH, estradiol, prolactin hormones were low, below the reference value for phase I of the menstrual cycle (<10 pg/mL). Women with subthalamic-pituitary functional disorders and PCOS (polycystic ovary syndrome), diagnosed based on the Rotterdam criteria, were enrolled in Group II [46]. In group V and VI, prolactinoma was diagnosed in one woman, and in another, functional hyperprolactinemia.

The patients were classified not only on the basis of hormonal tests, but also the clinical and ultrasound examination. Chronic anovulation is a common symptom in menstrual dysfunction with low progesterone serum concentration.

Blood for hormonal tests was taken between the 3rd and 5th day of the menstrual cycle. Normal range for estradiol in proliferation phase of the cycle was 10–129 pg/mL. Normal range for prolactin was 109–480 U/L.

Women with no hormonal disorders (confirmed with laboratory tests), a history of tobacco, alcohol, drugs or smart drugs abuse, with a history of psychiatric treatment, past or present, or with no consent for the study, not cooperating, taking androgenic drugs or pregnant were excluded from the study.

The control group consisted of 61 randomly selected women aged 18–40 (mean 28.07 ± 6.21 years. Patients in the control group were qualified on the basis of their history which confirmed that they had no disorders in the field of female sex hormones. Criteria for exclusion from the control group also included consumption of stimulants, and pregnancy.

### 2.2. Clinical Tests Performed to Diagnose TMD

Each patient (females with hormonal disorders and the control group, n = 126) had their full medical history taken as well as routine dental history for TMD. The clinical examination and functional diagnostics of the masticatory organ were performed with axis I and II of the Diagnostic Criteria for Temporomandibular Disorders (DC/TMD) [21]. These criteria cover TMD types which include pain-related types (e.g., myalgia, TMD-related headaches, and TMJ pains) as well as TMJ disorders (articular disc dislocation and degenerative disease). The examination, performed in accordance with the algorithm of the DC/TMD questionnaire, formed the basis for assigning the patients’ symptoms to one or many TMD. All examiners revised the special instructions for the examination (Specifications for DC/TMD Examination). The identification and confirmation of pain locations were established based on complaint and examination findings of familiar pain. If the patient reported “pain” during the examination procedures, the examiner asked the patient if the pain was familiar to them and the word “familiar” was always associated with the pain complaint outside the clinical setting. For DC/TMD pain diagnoses, the pain is relevant if it occurs within the period of the past 30 days. The vertical and horizontal incisal overlap and midline deviation were measured. Jaw-opening pattern and familiar pain were also assessed with jaw mobility testing. If the opening pattern was straight, there was no, or minimally, perceptible deviation (<2 mm) upon opening. Corrected or uncorrected deviation (right or left) was recorded. Additionally, the familiar headache in the temporalis was assessed with jaw mobility testing. The TMJ evaluation consisted of assessment of TMJ clicking, fine and coarse crepitus, patient-reported TMJ noise during movement, pain, and familiar pain associated with the click. Prior to palpation, the examiners were calibrated to use the specified forces. The patient was instructed to point with one finger to all of the areas that manifested pain. The following muscles and areas were palpated: the temporalis and masseter muscles, the posterior and submandibular muscles, lateral pterygoid areas, temporalis tendon, the lateral pole of the TMJ, and around the lateral pole of the TMJ. The assessment also included the familiar pain with palpation and familiar headache in the temporalis muscle with palpation. Millimeter measurements of opening, protrusive, and lateral excursive movements were recorded. The measurement was taken between the incisal edges of the maxillary and mandibular reference teeth. Pain-free opening and maximum unassisted opening was patient-based, while maximum assisted opening was examiner-based. Based on the subjective and objective examination according to DC/TMD, a specific diagnosis was established using the Diagnostic Decision Tree (Pain-related TMD—myalgia, arthralgia, and Headache; Intra-articular Joint Disorders—disk displacement with or without reduction, with intermittent locking, with or without limited opening, and Degenerative Joint Disorders).

On axis I, the condition of the masticatory muscles and the TMJ were examined. On axis II, the patients self-assessed their emotional condition (e.g., anxieties, apprehension, negativity, depression, psychosomatic diseases) and indicated the presence of chronic pain or discomfort in the area of the head, the neck and the joint itself (Pain Drawing, Graded Chronic Pain Scale).

All the subjects had given their informed consent for the inclusion in the study before actual participation. The study was conducted in accordance with the Declaration of Helsinki, and the protocol was approved by the Bioethics Committee of the Medical University of Warsaw (No 191/2017).

### 2.3. Statistical Analysis

Descriptive and numerical statistics and most calculations were performed by means of the Statistica 12 package. In statistical calculations, the significance level was set at *p* ≤ 0.05.

## 3. Results

Out of the 65 patients from the study group, as many as 60 (92.3%) were diagnosed with functional disorders within the masticatory organ. The most common ones included intra-articular conditions (articular disc displacement with and without reduction (DDwR, DDw/oR), subluxation), which were confirmed in 41 patients (63.07%). These were followed by myalgia (local myalgia, myofascial pain, MFP), reported by 29 patients and accounting for almost half of the participants (44.62%). TMJ degenerations (DJD), observed ineight patients (12.3%), were another TMD complaint.

Most of the patients with gynecological ailments were hospitalized due to hypothalamic pituitarism (group II of menstrual disorders acc. to WHO classification) (Table 1). The patients in this group included those with eating disorders (malnutrition or obesity) or ones with a tendency to “overexercise”, who demonstrated excessive physical activity (e.g., those who practised competitive sports, or frequented the gym). In this group of menstrual disorders correlated with physical activity, dysfunctions were manifested with pain in the masticatory organ. Complaints included spontaneous facial and masticatory muscle pain on eating, and morning stiffness of the facial muscles. These manifestations were reported by 92% of patients. The correlation between the muscular dysfunction of the masticatory organ with group II of menstrual disorders was significant (*p* = 0.0001).

Thirty percent of patients with hypothalamic pituitarism also experienced intra-articular disorders (*p* = 0.02). This group included patients in whom clinical examination and laboratory tests confirmed the diagnosis of PCOS. In this group of patients, intra-articular disorders were dominant, affecting 88% of women. A direct correlation between PCOS and intra-articular joint disorders was demonstrated (*p* = 0.005).

The next group of gynecological patients belonged to group III of menstrual disorders acc. to WHO classification—primary ovarian insufficiency. As many as 90% of women with these ailments were also diagnosed with TMJ intra-articular structure dysfunctions. In 30%, symptoms of degenerative TMJ were observed. A correlation between primary ovarian insufficiency and DJD was confirmed at *p* = 0.014 and with intra-articular dysfunctions at *p* = 0.005.

In another group of menstrual disorders, namely hypogonadotropic hypogonadism (group I of menstrual disorders acc. to WHO classification), the relevant patients were diagnosed with the clinical dysfunction of the masticatory organ related to internal derangement (DDwR, DDw/oR) and with DJD (22% of patients in this group of gynecological conditions (*p* = 0.05)).

The age distribution in the hormonal disorders group and in the controls was examined with the Shapiro-Wilk test. Due to the age of the patients in total and in the groups (hormonal disorders and controls), this did not have a normal distribution, or showed a clear asymmetry, the groups were more or less equal, and the comparison of the age in the groups was made using the parametric Student’s *t*-test for independent groups. The analysis of the material did not reveal statistically significant differences in the mean age of the hormonal disorders group and the controls (*p* = 0.953), (Table 2). In the control group, out of 61 patients, 55.73% had different types of TMD dysfunction, but their frequency in comparison to the hormonal disorders group (92.3%) was significantly lower (*p* = 0.021), mainly in the intra-articular disorders. The statistical study did not show any significant differences in the frequency of MFP between the groups (*p* = 0.519). Statistical analysis confirmed the significant differences in the occurrence of intra-articular disorders (DDwR and DDw/oR) in the hormonal disorders group and controls (*p* = 0.000) ( 63.07% and 18.03% respectively). It was observed that DDwR was more common in the hormone disorders group than in the controls (*p* = 0.0001). In addition, the DDw/oR was observed much more often in the hormonal disorders group compared to the controls (*p* = 0.01). When comparing the incidence of DJD in both groups, it was observed that degenerative changes occurred more frequently in the hormonal disorders group than in the control group, but these differences were not statistically significant (*p* = 0.142).

## 4. Discussion

Beside their influence on the female reproductive system, estrogens can affect the functioning of the whole body in multiple ways [13]. Estrogens, with their effect on the limbic system (responsible for behavior and emotional status), lower pain’s the threshold of pain and intensify its perception [47,48]. The occurrence of TMD is highly correlated with psychological factors—the patient’s personality traits. In women with temporomandibular joint disorders, the following manifestations have been observed: an increase in local symptoms and worse mental condition (stress, signs of depression, negativity, reduced ability to cope with stressful situations, suicidal tendencies) [19]. This relationship was also noted by the authors of the present study.

The fact that TMD concerns mostly women is an argument for the potential role of estrogens in their pathomechanism, peaking at the age of 20–45 years [14,16,19]. In the gestation period and during the menstrual cycle, hormonal fluctuations produce painful sensations in patients with TMD [42,43,49]. Fluctuations in estrogen levels at the childbearing age may intensify myofascial pain while the high level of estrogens in pregnant women predisposes to gingiva hyperplasia and periodontitis. In turn, low levels of estradiol during menopause contribute the development of TMJ degeneration, osteoporosis and alveolar bone resorption [17]. Women with TMD experience alleviation of painful sensations during pregnancy when the increase of pregnancy hormones is noted: progesterone, estrogens and relaxin, which have significant effect on degenerations in TMJ. Relaxin, which is produced only during pregnancy, increases flaccidity and mobility of TMJ, muscles, tendons and ligaments [43,50]. To confirm the role of progesterone in TMD etiopathogenesis, a clinical study was conducted in which increasing doses of progesterone were administrated for 10 days to female rats after ovariectomy, which resulted in alleviation of TMD pain. The authors made an optimistic presumption that progesterone could be prescribed in prevention and/or alleviation of pain in TMD [51]. The pathomechanism of influence of female reproductive hormones on masticatory organ is not fully known. 17-β estradiol (E2) plays a major role in the pathogenesis of the masticatory organ lesions in women of childbearing age; it induces the production of matrix metalloproteinase (MMP9 and MMP13) in the TMJ fibrocartilage and leads to its destruction [43,52,53,54]. Regrettably, the mechanisms of E2 destructive influence remain little recognized [48].

It is believed that estrogens affect mRNA replication and expression of Nav1.7 protein in the sodium channels of the trigeminal nerve ganglion, close these canals, and increase pain response by lowering the TMJ’s nociceptive threshold [15]. In female patients there is a larger number of estrogen receptors and their overexpression in the TMJ implicates excessive joint flaccidity. It is highly probable that the clinical picture is affected by estrogen receptors’ polymorphism [55]. The influence of estrogen receptor polymorphisms on TMD has been the subject of many studies. Dalewski et al. investigated the effect of ESR1 estrogen receptor polymorphism on the displacement of the articular disc without reduction compared to the control group and found that the genotype of the ESR1 predisposes to intraarticular disorders [56]. Moreover, Yu S et al. and Li F et al. suggest that a significant role can be played by estrogens produced locally in articular cartilage of the condylar process, bone, or synovial tissue, independently from estrogen’s level in the blood [57,58]. Estrogen deficiency may contribute to the development of lesions within the TMJ by restricting the synthesis of proteoglycans in the articular cartilage and collagen and proteins in the mandibular disc.

The analysis of the available reports in the literature indicates that the results of studies undertaken by different authors on the subject of the effect of the estrogens level on stomatognathic dysfunctions are inconsistent, and sometimes even contradictory. Berger et al. performed a systematic review of medical electronic databases (Medline/Pub Med, Scopus and Cochrane Library) and focused on nine papers, of which seven confirmed the correlation between TMD and the estrogen level [13]. Five of these papers implicated a low level of estrogens in the frequency and severity of painful sensations. On the other hand, there are reports which stress a positive correlation between the level of female reproductive hormones and the onset and frequency of TMD [59,60,61,62]. In the studies on rats, Bi RY et al. confirmed the hypothesis that 17-beta-estradiol might be responsible for excessive tenderness of the TMJ and the development of inflammatory processes [51]. On the basis of their results, Landi et al. suggested a possible correlation between a high estrogen level and the increased incidence of TMD with predisposition to develop degenerative disease of the TMJ [63,64]. TMD most often coexist with other systemic diseases which can modulate the course of TMD. There are more severe symptoms observed in patients with comorbidities than with isolated TMD [65,66]. Abnormalities in female reproductive hormone levels correlate with the frequency of TMD, intensify their symptoms, extend their duration or hinder the effectiveness of treatment. Yasutoka et al. and Cheng et al. investigated the effect of estrogen deficiency on the TMJ in ovariectomized rats, and observed the development of structural lesions such as decreased volume and density of bones, increased cartilage thickness and the occurrence of degenerative lesions [59,67].

Studies of patients on oral contraceptives (OCs), when the level of exogenous hormones is constant and lower than the lowest level of endogenous hormones, indicate a decisively higher perception of pain and prolonged treatment of these patients [60]. The authors suggest that low estrogen levels may contribute to the intensification of pain and therapeutic difficulties. Other conclusions were made by Lora VR et al. who stated that taking OCs was not linked to TMD and suggested no impact of exogenous hormones on the pain threshold [68], while the authors of the present study also observed a high percentage (92.3%) of TMD comorbidities in patients with a low level of estrogens, confirming previous studies [61]. The authors also noted domination of intra-articular dysfunctions (63.07%) and MFP (44.62%), although in the control group without sex hormone disorders more than a half of females (55.73%) had different types of TMD dysfunction, but the frequency was still significantly lower (*p* = 0.021). This difference concerned mainly intra-articular diseases (which predominated in patients in the I, II and III groups of menstrual disorders according to WHO) (*p* = 0.000). On the other hand, MFP and DJD concerned patients of both groups to a similar extent; although statistically insignificantly, they were more often observed in women with menstrual disorders.

In patients with functional hypothalamic pituitarism, in whom the level of female sex hormones (estrogens and progesterone) was low or proper (confirmed with clinical and laboratory tests), the MFP coexisted most often. In turn, in patients of other menstrual disorder groups according to WHO, with low level of estrogens and progesterone, in such conditions as polycystic ovarian syndrome (PCOS), primary ovarian insufficiency, organic hypothalamic-pituitary disorders, hyperprolactinemia, the intra-articular disorders prevailed (DDwR or DDw/oR). Heretofore, it has not been possible to examine fully the pathomechanism of female reproductive hormones in TMD. However, there is a high probability of the impact of polymorphism of its receptors on the clinical picture of TMD [69].

Interestingly, the present study revealed the occurrence of MFP in women with diagnosed hypothalamic-pituitary dysfunction, which was not diagnosed in patients with a different background of menstrual disorders. This requires further research and analysis of the pathomechanism of MFP, the more so as other types of menstrual disorders were associated with decreased estrogen levels. It is quite likely that the differentiating factor is the level of progesterone or the estrogen receptor polymorphism mentioned above. Another noteworthy element is the occurrence of DJD in women with diagnosed primary ovarian insufficiency characterized by reduced estrogen levels, primary or secondary amenorrhea, and infertility. It is necessary to take a broader look at the problem from the hormonal perspective, not only related to the level of female hormones but also the functioning of the pituitary gland and the level of thyroid hormones.

Considering the limitations of this study, such as the lack of hormone level analysis and the diagnosis of TMD based only on the DC/TMD clinical test, the indication that TMD may coexist with menstrual disorders is of utmost important. In TMD diagnostics and therapy, one has to consider systemic comorbidities, whose presence may modulate not only the onset of disorders and recurrence of ailments but also the effectiveness and course of treatment.

## 5. Conclusions

1. In women with menstrual disorders TMD may exist.
2. In women with TMD symptoms, their medical history should be extended to include the diagnosis of female hormone disorders.

## Figures and Tables

**Table 1:** Correlation between menstrual disorders and TMD (N = 65).

Menstrual Disorders According to WHO	N	Estrogen (E) and/or Progesterone (P) Levels	Clinical Examination	TMD According to DC/TMD	N	%	*p* = Value
I Group Hypogonadotropic hypogonadism	9	↓ E2	Amenorrhoea	Intra-articular joint disorders	9	100	0.0001
				Degenerative joint disease	2	22	0.05
II Group Disorders of hypothalamic pituitary axis	27	↓ or N E2 and P	Amenorrhoea or other menstruation disorders	Myalgia	25	92	0.0001
				Intra-articular joint disorders	8	30	0.02
				Degenerative joint disease	2	7	0.18
Group II Polycystic ovary syndrome (PCOS)	17	N E2	Amenorrhea or Sterility	Intra-articular joint disorders	15	88	0.005
				Myalgia	2	12	0.04
III Group Primary and secondary ovarian insufficency	10	↓E2	Amenorrhea or Sterility	Intra -articular joint disorders	9	90	0.005
				Degenerative joint disease	3	30	0.014
				Myalgia	2	20	0.02
Group IV	-	-	-	-	-	-	-
V and VI Group Hyperprolactinaemia	-	-	-	-	-	-	-
Prolactinoma	1	↓E2	Amenorrhoea	-	-	-	-
Functional hyperplolactinaemia	1	↓E2	Amenorrhoea or other menstruation disordres	Degenerative joint disease	1	100	0.001

*p* ≤ 0.05, Fisher exact test.

**Table 2:** Comparison of the frequency of TMD in females with hormonal disorders and the controls.

	N	Hormonal Disorders N/%	Control Group N/%	*p*
Number of women	126	65 (100%)	61(100%)	
Aged	18–40	28.00 ± 6.27	28.07 ± 6.21	0.953
TMD	94	60 (92.3%)	34 (55.73%)	0.021
MFP	53	29 (44.62%)	24 (39.34%)	0.519
Intra-articular disorders:	52	41 (63.07%)	11 (18.03%)	0.000
DDwR	39	30 (46,15%)	9 (14.75%)	0.000
DDw/oR	13	11 (16.92%)	2 (3.28%)	0.010
DJD	11	8 (12.3%)	3 (4.92%)	0.142

Chi-square test, *p* ≤ 0.05.

## Data Availability

Data supporting reported results can be found in dataset generated during this study.
